# Endothelial microvesicles in hypoxic hypoxia diseases

**DOI:** 10.1111/jcmm.13671

**Published:** 2018-05-29

**Authors:** Fan Deng, Shuang Wang, Riping Xu, Wenqian Yu, Xianyu Wang, Liangqing Zhang

**Affiliations:** ^1^ Department of Anesthesiology Affiliated Hospital of Guangdong Medical University Zhanjiang China; ^2^ Dongfeng General Hospital Hubei University of Medicine Shiyan China; ^3^ Department of Anesthesiology Taihe Hospital Hubei University of Medicine Shiyan China; ^4^ Institute of Anesthesiology Department of Anesthesiology Taihe Hospital Hubei University of Medicine Shiyan China

**Keywords:** biomarkers, endothelial microvesicles, hypoxia, ischaemic

## Abstract

Hypoxic hypoxia, including abnormally low partial pressure of inhaled oxygen, external respiratory dysfunction‐induced respiratory hypoxia and venous blood flow into the arterial blood, is characterized by decreased arterial oxygen partial pressure, resulting in tissue oxygen deficiency. The specific characteristics include reduced arterial oxygen partial pressure and oxygen content. Hypoxic hypoxia diseases (HHDs) have attracted increased attention due to their high morbidity and mortality and mounting evidence showing that hypoxia‐induced oxidative stress, coagulation, inflammation and angiogenesis play extremely important roles in the physiological and pathological processes of HHDs‐related vascular endothelial injury. Interestingly, endothelial microvesicles (EMVs), which can be induced by hypoxia, hypoxia‐induced oxidative stress, coagulation and inflammation in HHDs, have emerged as key mediators of intercellular communication and cellular functions. EMVs shed from activated or apoptotic endothelial cells (ECs) reflect the degree of ECs damage, and elevated EMVs levels are present in several HHDs, including obstructive sleep apnoea syndrome and chronic obstructive pulmonary disease. Furthermore, EMVs have procoagulant, proinflammatory and angiogenic functions that affect the pathological processes of HHDs. This review summarizes the emerging roles of EMVs in the diagnosis, staging, treatment and clinical prognosis of HHDs.

## 1 INTRODUCTION

Hypoxia is the lack of oxygen supply to tissues, which results in abnormal cell metabolism and function as well as pathological morphological alterations.^1^, ^2^, ^3^ The aetiology of hypoxia‐related diseases is complex and can be divided into four categories: hypoxic hypoxia induced by an arterial oxygen pressure drop, circulatory hypoxia caused by tissue blood flow reduction, hemic hypoxia induced by haemoglobin reduction and dysoxidative hypoxia caused by altered bio‐oxidation of tissues.^4^ Oxidative stress, inflammation and coagulation are closely related to the occurrence, development and treatment of hypoxia‐related diseases.

Hypoxic hypoxia is decreased arterial oxygen partial pressure resulting in tissue oxygen deficiency; the specific characteristics are reduced arterial oxygen partial pressure and oxygen content. The aetiologies and mechanisms of HHDs include abnormally low partial pressure of inhaled oxygen (eg plateau‐induced atmospheric hypoxia), external respiratory dysfunction‐induced respiratory hypoxia (eg obstructive sleep apnoea [OSA]) and venous blood flow into the arterial blood (eg in right to left shunt congenital heart disease and pulmonary hypertension).

Endothelial microvesicles are 100‐1000 nm anucleated vesicles formed following cytoskeletal and membrane reorganization and are released during apoptosis or activation of ECs into the extracellular milieu.^5^, ^6^ EMVs that are released from apoptotic or activated ECs that have been stimulated by hypoxia, oxidative stress, coagulation and inflammation can be used as a marker of EC injury. Coincidentally, elevated EMV levels have been identified in several HHDs,^7^ including OSA^8^ and pulmonary hypertension (PH).^9^ Furthermore, EMVs play a critical role in cell information transmission and exchange, and the procoagulant, proinflammatory and angiogenic properties of EMVs have been confirmed to increase in the occurrence and development of HHDs. All these findings indicate that EMVs have the potential to identify HHD phenotypes, to stratify disease severity, to improve risk stratification for patients who develop HHDs, to better define prophylactic strategies and to ameliorate the prognostic characterization of patients with HHDs. Furthermore, because a pathogenic role for EMVs is clearly emerging in HHDs, EMVs are becoming a novel target for HHD treatment.

## 2 ENDOTHELIAL MICROVESICLES

Endothelial microvesicles are 100‐1000 nm anucleated vesicles that are formed following cytoskeletal and membrane reorganization and can be released into the extracellular milieu following apoptosis or activation of ECs.^5^, ^10^ Hypoxia, ischaemia, oxidative stress, inflammation, coagulation and other factors can damage ECs or cause EC activation.^11^ Previous studies also found elevated EMV levels in several disease conditions associated with hypoxic hypoxia,^7^ such as OSA,^8^ chronic obstructive pulmonary disease (COPD)^12^ and PH.^9^ In addition, EMVs may play important roles in the pathological processes^13^ and tissue repair mechanisms^14^ of HHDs.

### 2.1 EMV phenotypes

As the surface of EMVs contains a variety of membrane glycoprotein antigens (such as CD31, CD144 and others), EMVs can be defined and detected using membrane glycoprotein antigens. These membrane glycoprotein antigens also make it easier to identify, distinguish and study EMVs.

**Figure 1:**
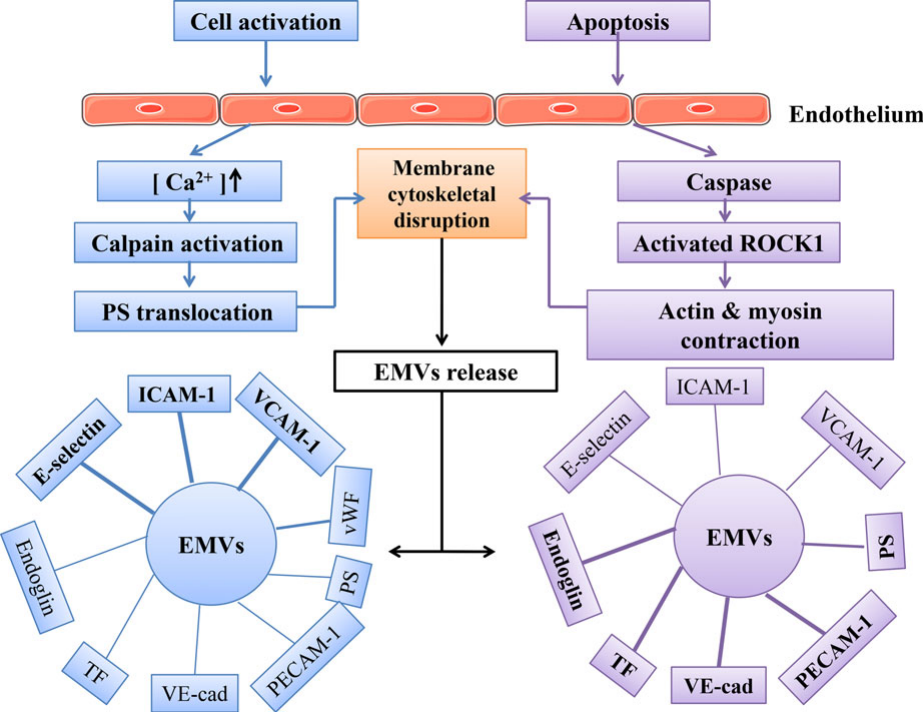
Differences in the release mechanism and antigen expression of EMVs derived from activation vs apoptosis of ECs. Activated stimuli cause a cytosolic calcium increase, which leads to EC membrane disruption. Apoptotic stimuli activate caspases and cause membrane disruption in ECs. Activation inducers and apoptosis inducers can both lead to vesiculation and EMV generation, but the levels of EMVs surface antigen markers are not the same. The surface of activated EMVs contains a higher level of E‐selectin, ICAM‐1, and VCAM‐1, while PS, PECAM‐1, VE‐cad, TF, and endoglin antigen markers show higher expression on apoptotic EMVs. ICAM, intercellular adhesion molecule; TF, tissue factor; VCAM, vascular cell adhesion molecule; PECAM, platelet endothelial cell adhesion molecule; VE‐cad, VE‐cadherin; PS, phospholipid; vWF, von Willebrand factor^10^, ^11^, ^17^, ^18^, ^19^, ^20^, ^21^

First, membrane glycoprotein antigens help us to identify EMVs. We can use membrane glycoprotein antigens to detect and identify EMVs. However, a major issue is that most surface markers are not unique to EMVs. In the blood, platelets, red blood cells and other cells can release microvesicles, and many different cell‐derived microvesicles have common surface markers. To identify EMVs and distinguish EMVs from other vesicles, researchers have proposed a combination of marker proteins derived from different vesicles to resolve these difficulties. For example, we can use positive EC markers (eg CD31 and CD144) in combination with the absence of platelet markers (CD41 or CD42b) to distinguish between EMV and platelet microvesicles.^15^ As shown in our previous report^16^ and in Figure 1, the existing CD markers on the surface of EMVs include CD31/platelet endothelial cell adhesion molecule‐1 (PECAM‐1), CD51/integrin‐alpha V (Integrin‐av), CD54/intercellular cell adhesion molecule‐1 (ICAM‐1), CD62E/endothelial‐selectin (E‐selectin), CD105/Endoglin, CD106/vascular cell adhesion molecule‐1 (VCAM‐1), CD144/vascular endothelial‐cadherin (VE‐cadherin) and CD146/melanoma cell adhesion molecule (MelCAM).

Second, classification via membrane glycoprotein antigens will facilitate the identification of stimulatory factors that cause EC activation or apoptosis. As shown in Figure 1,^10^, ^11^, ^17^, ^18^, ^19^, ^20^, ^21^ Ang II and other cell activation factors can increase EC release of CD62E^+^ EMVs, CD54^+^ EMVs, and CD106^+^ EMVs. In contrast, when the endothelium becomes functionally apoptotic, CD31^+^, CD51^+^ and CD144^+^ EMV levels are increased. The relative proportion of CD62E^+^/CD31^+^ EMVs, rather than absolute levels, distinguishes EMVs released by activated ECs from those derived from apoptotic ECs. A level of CD62E^+^/CD31^+^EMVs less than 1% indicates that most EMVs originated from apoptotic ECs, whereas 10% or more reflects an activated origin.^20^

Third, different surface markers likely play different roles in the pathogenesis of diseases.^22^ As shown in Table 1, different EMV subtypes may play a role in different HHDs. The level of CD31^+^ EMVs is related to the apnoea‐hypopnea index (AHI) in OSA patients^23^, ^24^, ^25^; mild COPD and emphysema in COPD patients^26^, ^27^; and hemodynamic severity, risk stratification and treatment effects in PH patients.^9^, ^28^, ^29^ In addition, CD31^+^ EMVs may play diverse roles in vascular biology by regulating platelet function, angiogenesis, T‐cell and B‐cell activation, EC permeability and transmigration across the endothelium.^30^, ^31^, ^32^, ^33^, ^34^, ^35^ Therefore, the released CD31^+^ EMVs likely reflect the apoptosis of injured ECs. CD51 is present on ECs, B lymphocytes, monocytes, macrophages and platelets.^36^ CD51^+^ EMVs may increase leucocyte homing and rolling and angiogenesis.^35^, ^37^ The LFA‐1/ICAM‐1 interaction is critical to the firm adhesion of T cells to the vascular endothelium of inflamed tissues and influences the diapedesis and migration of these adhered lymphocytes out of the vasculature directly into adjacent tissues on the ocular surface.^38^ CD62E is rapidly induced on activated ECs a few hours after inflammatory stimulation, and CD62E^+^ EMVs may increase the recruitment of leucocytes to the site of injury during inflammation.^30^, ^31^, ^32^, ^33^, ^34^, ^35^ In OSA patients, the level of CD62E^+^ EMVs was related to treatment effects of continuous positive airway pressure (CPAP) and AHI.^39^ In COPD patients, the level of CD62E^+^ EMVs was a good predictor of rapid forced expiratory volume in 1 second (FEV1) decline, severe COPD and hyperinflation.^12^, ^27^, ^40^ In PH patients, the level of CD62E^+^ EMVs was related to adverse clinical events and thromboembolic complications.^41^, ^42^ In venous thromboembolism (VTE) patients, the level of CD62E^+^ EMVs was related to inherited thrombophilia.^43^ Thus, CD62E^+^ EMV levels may reflect the degree of ongoing endothelial inflammation. Endoglin EMVs are CD105^+^ EMVs. The level of CD105^+^ EMVs is related to severity, EC survival and angiogenesis in PH patients.^44^, ^45^ The level of CD144^+^ EMVs is related to hemodynamic severity, pulmonary artery intima media thickness and right ventricular function.^9^, ^46^, ^47^ The release of CD144^+^EMVs may reflect the structural destruction of the endothelium rather than an inflamed lung. CD146^+^ EMVs, another indicator of EC injury, may be closely related to signal transduction, endothelial permeability, cell migration, angiogenesis and immune response.^48^

**Table 1:** The different EMVs related to diseases

EMVs	Diseases	Aspects related to diseases	References
CD31^+^	OSA	Accelerated atherosclerosis and increased cardiovascular risk	22
		Independent associations with the AHI	21, 23
	COPD	Mild COPD and emphysema	24, 25
	PH	Hemodynamic severity, risk stratification, treatment effects.	7, 26, 27
CD62E^+^	OSA	Treatment effects of CPAP, the AHI in children	37
	COPD	A predictor of rapid FEV1 decline, severe COPD and hyperinflation	11, 25, 38
	PH	Adverse clinical events, thromboembolic complications	39, 40
	VTE	Inherited thrombophilia	41
CD105^+^	PH	Severity of PAH, endothelial cell survival and angiogenesis	42, 43
CD144^+^	PH	Hemodynamic severity, pulmonary artery intima media thickness, right ventricular function	7, 44, 45

AHI, apnoea‐hypopnoea index; COPD, chronic obstructive pulmonary disease; CPAP, Continuous positive airway pressure; ECs, endothelial cells; FEV1, forced expiratory volume in 1 second; OSA, Obstructive sleep apnoea; PAH, pulmonary arterial hypertension; PH, pulmonary hypertension; VTE, venous thromboembolism.

### 2.2 EMVs as messengers

**Figure 2:**
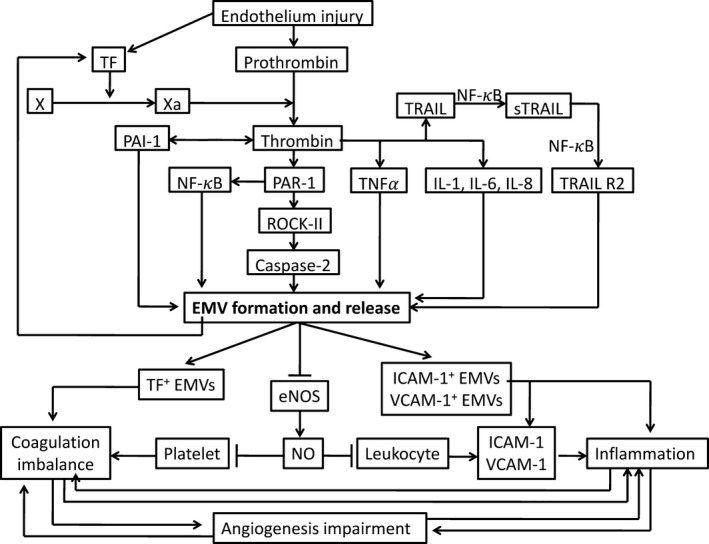
Signalling pathways involved in thrombin‐induced EMV formation and potential mechanisms involving circulating EMVs in the interrelationship among inflammation, angiogenesis and coagulation. Thrombin induces TNF‐alpha, IL‐1, IL‐6, IL‐8, PAR‐1, TRAIL and PAI‐1, which lead to formation of EMVs. EMVs carry TF, the major initiator of the extrinsic pathway of the coagulation cascade, and ICAM‐1 and VCAM‐1, which play an important role in vascular inflammation. In addition, EMVs circulating in plasma impair eNOS and subsequent release of NO, which leads to platelet adhesion, aggregation and prothrombotic events. Circulating EMVs might also favour vascular inflammation by promoting leucocyte adhesion through increased expression of ICAM‐1 and VCAM‐1 or impaired NO release. ICAM, intercellular adhesion molecule; TF, tissue factor; VCAM, vascular cell adhesion molecule; TNF‐a, tumour necrosis factor alpha; NF‐κB, nuclear factor kappa B; ROCK‐II, Rho kinase; PAR‐1, proteolytically activated receptor‐1; TRAIL, tumour necrosis factor‐related apoptosis‐inducing ligand; eNOS, endothelial nitric oxide synthase; NO, nitric oxide^17^, ^49^

As shown in Figure 1,^10^, ^11^, ^17^, ^18^, ^19^, ^20^, ^21^ EMVs can be used as an indicator to reflect stimulatory factors, the disease state and prognosis. In contrast, EMVs also play a role in pathological processes and disease development. EMVs have important functions in the inflammatory response, coagulation and angiogenesis. As shown in Figure 2,^17^, ^49^ we identified the signal pathways involved in thrombin‐induced EMV formation and potential mechanisms involving in circulating EMVs in the interrelationship among inflammation, angiogenesis, and coagulation. We previously published an article detailing these features of EMVs; thus, for more detailed information, please refer to our previously published paper.^16^

## 3 EMVS IN HHDS

Endothelial microvesicles have been investigated in several human HHDs, which include very low partial pressure of inhaled oxygen, external respiratory dysfunction‐induced respiratory hypoxia and venous blood flow into the arterial blood, as possible pathogenic elements, prognostic markers, and therapeutic targets. The data reported in the following sections are summarized in Table 2.

**Table 2:** The EMVs in hypoxic hypoxia diseases

Diseases	Changes of EMVs	Major finding	References
Atmosph–eric hypoxia	CD31^+^/AV^+^ EMVs ↑ & CD62^+^/AV^–^ EMVs ↓ & CD31^+^/AV^–^ EMVs ↔ & CD62^+^/AV^+^ EMVs ↔ (high sea level vs low sea level)	Temporary hypoxic conditions can trigger the release of the CD31^+^/AV^+^ EMVs also in healthy volunteers.	50
	CD31^+^/CD41b^–^ EMVs ↔(high sea level vs low sea level)	At high altitude (3800 m), the intervention (using a 30‐minute distal cuff occlusion) elicited a reduction in flow‐mediated dilatation; this reduction was inversely correlated with the change in CD31^+^/CD41b^–^EMVs.	51
	CD62^+^ EMVs↑ & CD144^+^ EMVs↑& CD31^+^ EMVs ↔(high sea level vs low sea level)	Atmospheric hypoxia is associated with increased oxidative stress and induces a significant increase in CD62^+^ EMVs and CD144^+^ EMVs, which indicate that endothelial activation rather than an apoptosis is the primary factor of atmospheric hypoxia‐induced endothelial dysfunction.	52
	CD62^+^/AV^+^ EMVs ↔ & CD31^+^/AV^+^ EMVs ↔ (high sea level vs low sea level); The concentration of CD31^+^/CD42^−^ EMVs↓ & CD31^+^/AV^–^ EMVs↓ & CD62^+^/AV^+^ EMVs ↔(high sea level vs low sea level)	Moderate atmospheric hypoxia at an altitude of 2978 m seems to have positive effects on EMVs as shown by a significant reduction of circulatory levels of EMVs.	54
	CD31^+^/CD41^−^ EMVs ↔ & CD62E^+^ EMVs ↔ & CD106^+^ EMVs ↔ & CD144^+^ EMVs ↔ (high sea level vs low sea level)	In healthy male individuals, mild atmospheric hypoxia, induced by a short‐term stay at moderate altitude, is not significantly change in EMVs.	53
OSA	CD31^+^/CD42b^–^ EMVs↑ (patients with AHI≥5 vs matched volunteers free of CVRFs)	These early EMVs alterations may underlie accelerated atherosclerosis and increased cardiovascular risk in OSA	24
	CD31^+^/CD41^−^ EMVs ↔ (patients with ODI4%≥7.5 vs matched volunteers ODI4%<5)		75
	CD146^+^ EMVs ↔ (patients with ODI3%≥10 vs matched volunteers ODI3%<10 and no CVRFs)		8
	CD31^+^/CD42^−^ EMVs ↑ & CD62E^+^ EMVs ↑ & CD31^+^/AV^+^ EMVs ↑ (patients with AHI≥5 vs controls with AHI<5);CD62E^+^ EMVs ↓ & CD31^+^/CD42^−^ EMVs ↔ & CD31^+^/AV^+^ EMVs ↔ (CPAP treatment vs controls)	The EMV level is correlated with AHI	25
	CD31^+^/CD42b^–^/AV^+^ EMVs↑& CD62E^+^/CD42b^–^/AV^+^ EMVs ↑ (childhood patients with AHI≥1 vs controls with AHI<1)	EMVs levels show independent associations with the AHI in childhood OSA patients.	23
	CD62E^+^ EMVs ↑ (the CPAP withdrawal group vs the continuing therapeutic CPAP group)	CD62E^+^ EMVs formation may be causally linked to OSA and may promote endothelial activation.	39
COPD	CD62E^+^ EMVs & CD144^+^ EMVs (patients under a stable condition had significant negative correlations with annual FEV1 changes)	The high CD62E^+^ EMV levels in stable patients with COPD are predictive of rapid FEV1 decline.	12
	CD31^+^/CD42b^–^/AV^+^ EMVs ↑ & CD31^+^/CD62E^+^ EMVs ratios ↑ (patients with normal spirometry but reduced D_LCO_ vs controls)	The early development of emphysema in COPD might be monitored with plasma EMV levels.	26
	CD31^+^ EMVs ↑ (severe COPD/ mild COPD vs controls); CD62E^+^ EMVs ↑ (severe COPD vs controls);CD51^+^ EMVs ↔ (severe COPD/ mild COPD vs controls)	CD31^+^ EMVs, suggestive of endothelial cell apoptosis, were elevated in mild COPD and emphysema. In contrast, CD62E^+^ EMVs indicative of endothelial activation were elevated in severe COPD and hyperinflation.	27
	CD144^+^ EMVs ↑ & CD31^+^ EMVs ↑ & CD62E^+^ EMVs ↑ (stable patients vs healthy controls; patients with exacerbated COPD vs stable COPD patients);CD62E^+^ EMVs ↑ (patients with frequent exacerbations vs those with no frequent exacerbations)	Endothelial damage, mainly in pulmonary capillaries, occurs during exacerbation and continues even after clinical symptoms disappear. Higher baseline CD62E^+^ EMV levels may indicate COPD patients who are susceptible to exacerbation.	40
PH	CD31^+^ EMVs ↑ & CD144^+^ EMVs ↑ & CD62E^+^ EMVs ↑(patients vs controls)	The levels of CD31^+^ EMVs and CD144^+^ EMVs, but not CD62E^+^ EMVs, predicted hemodynamic severity in PH patients.	9
	CD105^+^ EMVs ↑ (PAH patients vs controls; pulmonary arterial blood in PAH patients vs venous blood in PAH patients)	Circulating CD105^+^ EMVs appear to be valuable tools in determining the severity of PAH.	44
	CD62E^+^ EMVs ↑(patients vs controls)	Elevated levels of circulating CD62E^+^ EMVs in PH patients prior to treatment are associated with adverse clinical events.	41
	CD144^+^ EMVs ↑& CD146^+^ EMVs ↑ (patients vs controls)	The levels of CD144^+^ EMVs are positively significant to pulmonary artery intima media thickness, but not CD146^+^ EMVs.	46
	CD31^+^/CD41^−^ EMVs ↔ (irreversible PAH vs reversible PAH)		28
	CD144^+^/AV^+^ EMVs ↑ in urine (patients vs controls)	Urinary CD144^+^/AV^+^ EMVs may be useful as potential biomarkers of right ventricular function in PAH.	47
	CD105^+^ EMVs ↑ (CTEPH vs healthy/disease controls)	Increased generation of CD105^+^ EMVs in CTEPH is likely to represent a protective mechanism supporting endothelial cell survival and angiogenesis, set to counteract the effects of vascular occlusion and endothelial damage.	45
	CD62E^+^ EMVs ↑ (patients vs healthy controls; patients with a thromboembolic PH vs non‐embolic PH patients)	CD62E^+^ EMVs indicating an increased vascular procoagulation and inflammation which might be related to thromboembolic complications as well as PH progression.	42
	CD31^+^/CD42b^–^ EMVs ↓ (PAH patients with human hepatocyte growth factor treatment vs controls)	Transfer of human HGF may attenuate the inflammatory cell infiltrate, reduce the expression of inflammatory factors, and those effects are possibly due to the inhibition of EMV production which may decrease pulmonary vascular wall damage in PAH.	29
ARDS	CD54^+^/AV^+^ EMVs ↑ (lipopolysaccharide induced ARDS in rats vs controls)		72
VTE	CD31^+^/CD42b^–^ EMVs ↑ & CD62E^+^ EMVs ↑ & EMV‐M ↑(patients vs controls)	The release of EMV and their binding to monocytes are key events in thrombogenesis.	68
	CD62E^+^ EMVs ↑ (patients vs controls)	Elevated levels of circulating CD62E^+^ EMVs can play a role in carriers of mild and severe inherited thrombophilia.	43
	CD62E^+^ EMVs ↑ (patients with PTM carriers and a previous VTE history vs controls)		70
	CD62E^+^ EMVs ↑ (patients with previous VTE vs controls without VTE)	Circulating CD62E^+^ EMVs may contribute to the development of VTE in carriers of factor V Leiden (FVL) mutation.	69
PE	CD31^+^ EMVs ↔ (patients vs healthy control subjects with no history of venous thromboembolism or vascular risk factors)		71

↑, increased; ↓, decreased; ↔, unchanged; AHI, apnoea–hypopnoea index; AV, annexin V; COPD, chronic obstructive pulmonary disease; CPAP, Continuous positive airway pressure; CTEPH, chronic thromboembolic pulmonary hypertension; CVRF, cardiovascular risk factor; D_LCO_, diffusing capacity of the lung for carbon monoxide; ECs, endothelial cells; EMP‐M, Endothelial microparticles‐ monocytes aggregates; EMP‐P, Endothelial microparticles‐platelets aggregates; FEV1, forced expiratory volume in 1 second; ODIn%, n% oxygen desaturation index; OSA, Obstructive sleep apnoea; PAH, pulmonary arterial hypertension; PH, pulmonary hypertension; PTM, prothrombin gene mutation G20210A; VTE, venous thromboembolism.

### 3.1 EMVs and atmospheric hypoxia

When people are at an altitude of 3000 metres above sea level or at a high altitude, are in poor ventilation tunnels or inhale a hypoxic mixture, the amount of oxygen in the body first depends on the oxygen partial pressure of the inhaled gas. In a plateau, as the altitude increases, the atmospheric pressure decreases, and the partial pressure of oxygen of the inhaled gas decreases correspondingly, which results in a decrease in the partial pressure of oxygen and oxygen in the alveoli. Then, the oxygen diffused into the blood decreases, and the arterial oxygen saturation decreases. Lichtenauer et al. found that circulating CD31^+^/Annexin V (AV)^+^ EMVs were correlated with an increase in simulated sea level and decline in oxygen saturation in young healthy volunteers. Concomitantly, we found a significant decrease in CD62^+^/AV^−^ EMVs when the partial pressure of oxygen declined with increasing sea level.^50^ We believe that this difference is attributable to CD31^+^ EMVs (primarily released from apoptotic ECs and reflecting hypoxia‐induced acute injury, while CD62E^+^ EMVs are primarily released from activated ECs). As Table 2 shows, however, the results of different groups are not consistent or even contradictory. This difference may be closely related to the altitude selected by the researchers.^51^, ^52^, ^53^, ^54^

### 3.2 EMVs and OSA

Obstructive sleep apnoea, including intermittent hypoxia and intrathoracic pressure changes and arousals, can result in endothelial dysfunction and ultimately arterial disease.^39^, ^55^, ^56^ There is growing evidence that EMVs can be used as a biomarker for diagnosis, treatment and prognosis in patients with OSA. Significantly, higher levels of EMVs have been reported, both in adult and childhood OSA, compared with matched controls. In addition, EMVs were correlated with OSA severity and the AHI.^23^, ^24^, ^25^, ^39^ Moreover, injection of EMVs from OSA patients into mice impaired the endothelium‐dependent relaxation in the aorta and FMD in small mesenteric arteries.^8^ Therefore, circulating EMVs are considered not only a marker of vascular damage but also a cause of vascular disease in patients with OSA. This relationship is one mechanism that links OSA and accelerated atherosclerosis, and increased levels of circulating EMVs may underlie the altered endothelial function and coagulation homeostasis in OSA.^24^, ^57^ However, 2 research groups found no increase in CD31^+^/CD41^−^ or CD146^+^ EMVs in OSA patients, which may be due to differences in EMV subpopulations and population differences.^8^, ^58^

Continuous positive airway pressure treatment improves endothelial function in OSA patients.^57^, ^59^, ^60^ Following CPAP treatment, CD62E^+^ EMVs (but not CD31^+^/CD42^−^ EMVs) were significantly reduced.^25^ These findings were also confirmed by Jelic et al.^24^, who reported elevated levels of EMVs in OSA patients and a trend towards decreased levels following CPAP in a treatment uncontrolled study. In addition, CPAP withdrawal leads to OSA recurrence, and CD62E^+^ and CD31^+^/CD42b^–^ EMV levels were increased significantly in the CPAP withdrawal group compared with patients administered continuous therapy.^39^

In addition, the ratio of CD62E^+^ EMVs to CD31^+^ EMVs, which contributes to distinguishing between activation and apoptosis ECs, suggests that EMVs in children and adults with OSA are primarily the apoptotic subtype. As markers of EC apoptosis, CD31^+^/CD42b^–^ EMV levels were strongly correlated with OSA severity, endothelial dysfunction and carotid intima media thickness^23^, ^25^ and may reflect the chronic vascular damage induced by long‐term exposure to repeated apnoeas. In contrast, CD62E^+^EMV levels (reflecting EC activation) are not correlated with OSA or the severity of vascular damage but are correlated with CPAP initiation or withdrawal.^15^, ^25^, ^39^

### 3.3 EMVs and Asthma

Asthma is usually characterized by different patterns of airway inflammation with a complex network of cellular and molecular mediators.^61^ In contrast to OSA, very little is known about the possible role of EMVs in asthma. The scarce available data are preliminary, and further research in the field with properly designed studies is needed.

### 3.4 EMVs and COPD

Chronic obstructive pulmonary disease is a lung disease characterized by nearly irreversible lung destruction, which results in airflow limitation. Accumulating evidence suggests that EC injury in lung tissues is closely related to disease progression in COPD.^62^, ^63^ These close associations might be mediated by both endothelial dysfunction and activation, indicating an active pathogenic role for EMVs, which can be a marker of COPD‐related clinical symptoms. Takahashi et al.^12^ found that the levels of CD62E^+^ EMVs and CD144^+^ EMVs showed a significant negative correlation with annual FEV1 changes, while high CD62E^+^ EMV levels in stable COPD patients were predictive of a rapid FEV1 decline. Furthermore, Gordon et al.^26^ demonstrated that EMVs could represent a potential marker of early emphysema; a low ratio of CD62E^+^ to CD31^+^ EMVs indicates a high percentage of apoptotic EMVs in early emphysema, while the presence of angiotensin‐converting enzyme on the surface of most EMVs indicates a pulmonary capillary origin.

In addition, many scholars have confirmed that EMVs can help to identify COPD phenotypes and stratify disease severity. Takahashi et al.^22^ found that CD31^+^ EMVs, CD62E^+^ EMVs and CD144^+^ EMVs were significantly increased in stable COPD patients and during exacerbation compared with healthy control subjects; meanwhile, circulating CD146^+^ EMVs were not increased in stable COPD patients or during exacerbation. Furthermore, most circulating EMVs during exacerbation were shown to be negative for von Willebrand factor (vWF) (a marker of systemic vasculature not expressed by pulmonary capillaries), suggesting they primarily originated from pulmonary capillary ECs.^22^, ^40^ Thomashow et al. found that CD31^+^ EMVs, suggestive of EC apoptosis, were elevated in mild COPD and emphysema patients compared with control subjects. In contrast, CD62E^+^ EMVs indicative of endothelial activation were elevated in patients with severe COPD and hyperinflation, which could predict susceptibility to exacerbations and represent a possible prognostic marker. Thus, EC apoptosis occurs early in the pathogenesis of COPD and emphysema, while endothelial activation is found in severe, hyper‐inflated COPD.^27^

### 3.5 EMVs and PH

Vascular remodelling and endothelial dysfunction‐related EMVs are involved in PH.^64^ Three research groups have demonstrated that EMV levels were significantly higher in PH patients than controls. The levels of CD31^+^ EMVs and CD144^+^ EMVs, but not those of CD62E^+^ EMVs, predict hemodynamic severity in PH patients.^9^ Elevated levels of circulating CD62E^+^ EMVs in PH patients prior to treatment were associated with adverse clinical events.^41^ The levels of CD144^+^ EMVs were positively associated with pulmonary artery intima media thickness but not CD146^+^ EMVs.^46^ Furthermore, Bakouboula et al. found differences in EMVs between the occluded pulmonary artery and the jugular vein. CD105^+^ EMVs were elevated in patients with pulmonary arterial hypertension (PAH) compared with those in control subjects, with a further increase in CD105^+^ EMVs observed in pulmonary arterial blood compared with venous blood in patients with PAH.^44^ In addition, Rose et al. found that urinary CD144^+^/AV^+^ EMV levels were increased in PAH patients compared with controls and that CD144^+^/AV^+^ EMVs were directly correlated with tricuspid annular plane systolic excursion (TAPSE) in PAH patients.^47^ Therefore, urinary CD144^+^/AV^+^EMVs may be useful as potential biomarkers of right ventricular function in PAH. However, M. Smadja et al. found no significant difference in CD31^+^/CD41^−^EMV levels between irreversible and reversible PAH.^28^

Belik et al. demonstrated that CD105^+^ EMV levels were significantly higher in chronic thromboembolic pulmonary hypertension (CTEPH) patients than in pulmonary embolism (PE) patients and healthy controls.^45^ Increased generation of CD105^+^ EMVs in CTEPH likely represents a protective mechanism supporting EC survival and angiogenesis that counteracts the effects of vascular occlusion and endothelial damage. Consistently, Diehl et al. found that CD62E^+^ EMVs were elevated in PH patients compared with those in control subjects, and a further increase in CD62E^+^EMVs was observed in thromboembolic PH patients compared with those in non‐embolic PH patients.^42^ CD62E^+^ EMVs reflect increased vascular procoagulation and inflammation, which might be related to thromboembolic complications and PH progression.

Endothelial microvesicles can be used as indicators of prognosis. Chen et al. found that the levels of inflammatory factors and EMVs were significantly lower in PAH patients with human hepatocyte growth factor (HGF) gene transfer treatment than in controls. Transfer of human HGF may attenuate inflammatory cell infiltration and reduce the expression of inflammatory factors, effects possibly due to reduced EMV production, which may decrease pulmonary vascular wall damage in PAH.^29^

### 3.6 EMVs and acute lung injury/acute respiratory distress syndrome

Many studies have indicated that acute lung injury (ALI)/acute respiratory distress syndrome (ARDS) could induce the release of different EMVs, which may serve as potential biomarkers and contribute to lung injury severity in ALI/ARDS patients.^65^, ^66^ Released EMVs impair vascular function by initiating coagulation,^67^ attenuating nitric oxide (NO) production from ECs,^68^, ^69^ stimulating inflammatory cytokine release and disrupting EC barrier integrity. EMVs derived from plasminogen activator inhibitor‐challenged ECs may contribute to ALI/ARDS development via increased cytokine production and neutrophil recruitment.^70^ EMVs induced by plasminogen activation inhibitor‐1 (PAI‐1) contribute to lung injury by initiating a cytokine cascade that increases neutrophil recruitment and a subsequent release of myeloperoxidase (MPO). Furthermore, treatment of mice with both EMVs and lipopolysaccharide (LPS) induces greater lung injury than either treatment alone, suggesting that EMVs prime the lung for increased injury by other pathogens. These findings demonstrated that EMVs are capable of inducing significant lung injury at pathophysiologically relevant levels.^69^ The role of EMVs in inducing ALI/ARDS and endothelial dysfunction may present new therapeutic targets.^70^ Finally, in a murine model, the injection of EMVs into mice induced a significant release of the proinflammatory cytokines TNF‐α and IL‐1β and a subsequent recruitment of neutrophils.^70^ These effects were further increased by the concomitant or sequential administration of bacterial LPS, indicating that EMVs might represent a signal that primes the lung for the following inflammatory response to an external injury.^71^ Again, from this point of view, EMVs could represent a potential therapeutic target for ALI/ARDS. EMVs could be a link between alveolar inflammation and coagulation (2 key steps in the pathobiology of ALI/ARDS) and may be a target for future treatment.

### 3.7 EMVs and venous thromboembolism/pulmonary embolism

The pathophysiology of VTE involves endothelial damage, blood stasis and hypercoagulability.^72^ EMVs may be used as diagnostic and differential diagnostic criteria in VTE and PE. EMV levels were significantly higher in patients with VTE,^43^, ^73^ a previous VTE history,^74^, ^75^ and the prothrombin gene mutation G20210A (PTM) compared with controls.^74^ This finding implies that elevated levels of circulating CD62E^+^EMVs act as carriers of mild and severe inherited thrombophilia and may contribute to the development of VTE in factor V Leiden (FVL) mutation carriers. In addition, EMVs are also involved in disease pathophysiology. Chirinos et al. found that patients with VTE showed marked elevation of EMV generation and EMV‐monocyte conjugates.^73^ These findings indicate that EMV release and binding to monocytes are key events in thrombogenesis.

However, Bal et al. found that circulating procoagulant MV levels were significantly higher in acute PE patients than in healthy control subjects with no history of VTE or vascular risk factors.^76^ Meanwhile, CD31^+^ EMV levels were not significantly changed.^76^ This discrepancy may be due to population and EMV subpopulation differences.

## 4 CIRCULATING EMVS WITH DISTINCT MICRORNAS CARGO IN HHDS

MicroRNAs (miRNAs) are a class of small non‐coding RNAs that work in regulation of gene expression by binding 3′untranslated region of target mRNAs. The miRNAs control cellular activities during cell growth, differentiation, apoptosis adhesion and cell death.^77^ Circulating EMVs with distinct miRNAs cargo have been detected in different diseases, which indicated circulating miRNAs in EMVs may be emerging as biomarkers of HHDs.^78^

Lars Eichhorn et al. found that a time‐dependent increase of circulating EMVs which carry different miRNAs in apnoea and circulating miR‐126 levels were elevated at all time‐points after a single voluntary maximal apnoea, whereas miR‐26 levels were elevated significantly only after 30 minutes and 4 hours. Also, miR‐21 and miR‐92 levels increased, but did not reach the level of significance.^79^ Serban et al. found that circulating EMVs are emerging as biomarkers of COPD in individuals exposed to cigarette smoke and the EMVs were significantly enriched in let‐7d, miR‐191, miR‐126 and miR125a, which reciprocally decreased intracellular in cigarette smoke‐exposed endothelium.^80^ Furthermore, the results indicate that cigarette smoke releases circulating EMVs with distinct miRNA cargo and that EMVs affect the clearance of apoptotic cells by specialized macrophages, which may be important in the pathogenesis of diseases linked to endothelial injury and inflammation in COPD smokers.^80^

Although we have opened the mysterious veil of circulating miRNAs in EMVs, there is no doubt that we only know the tip of iceberg about EMVs with distinct miRNA cargo in HHDs.

## 5 CONCLUSIONS

Although the roles of EMVs in the development of clinical disorders are still unclear and the potential causal relationships between EMV release and particular disease processes are still being elucidated, there is little doubt that EMVs are good biomarkers for identifying HHD phenotypes, assessing disease severity, improving risk stratification for patients developing HHDs to better define prophylactic strategies, and allowing better prognostic characterization of HHD patients.

## ACKNOWLEDGEMENT

This review was supported by grants from National Natural Science of China (NSFC, 81270196, 81470405) to Dr. Zhang, Liangqing.

## CONFLICT OF INTEREST

Authors declare that they have no conflict of interest.
